# Acute ST-Segment Elevation Myocardial Infarction Following Multimodality Cancer Therapy in a HER2-Positive Breast Cancer Patient: A Case Report

**DOI:** 10.3390/jcdd13080392

**Published:** 2026-08-14

**Authors:** Jiani Dai, Guohui Chen, Yabin Liu, Yongli Ji, Liangliang Jia

**Affiliations:** 1 Department of Cardiology, The Second Affiliated Hospital, Zhejiang University School of Medicine, Hangzhou 310009, China; 22418420@zju.edu.cn (J.D.); chenguohui@zju.edu.cn (G.C.); liuyabin0916@zju.edu.cn (Y.L.); 2 Department of Endocrinology, The Second Affiliated Hospital, Zhejiang University School of Medicine, Hangzhou 310009, China; jiyongli@zju.edu.cn

**Keywords:** human epidermal growth factor receptor 2, breast cancer, myocardial infarction, cancer therapy, cardiovascular toxicity, cardio-oncology

## Abstract

Cardiovascular toxicity associated with anti-tumor therapies is garnering increased attention. Anti-tumor therapies may elevate the risk of acute coronary syndrome, particularly in patients with underlying subclinical atherosclerosis. In this context, a 58-year-old female patient with HER2-positive breast cancer experienced an acute ST-segment elevation myocardial infarction following chemotherapy, dual HER2-targeted therapy, and radiotherapy to the left breast. Prior to the initiation of anti-tumor treatment, chest-computed tomography had identified coronary artery calcification. Angiographic evaluation revealed subtotal occlusion of the middle segment of the left circumflex artery extending to the first obtuse marginal branch, accompanied by multivessel coronary atherosclerotic lesions. Coronary blood flow was restored following emergency interventional treatment. This case indicates that multimodal anti-tumor therapy may facilitate the progression of coronary atherosclerosis, thereby elevating the risk of acute coronary events.

## 1. Introduction

Cardiovascular toxicity associated with anti-tumor therapies has emerged as a significant concern in the long-term management of patients with breast cancer [1]. Current research on cardiotoxicity related to human epidermal growth factor receptor 2 (HER2) predominantly addresses left ventricular dysfunction and heart failure, with comparatively less focus on vascular toxicity and ischemic cardiovascular events [2]. Increasing evidence indicates that HER2-targeted therapies, taxanes, and chest radiotherapy may contribute to vascular endothelial injury and the progression of atherosclerosis [3,4,5,6]. Multimodal anti-tumor treatments, in conjunction with pre-existing cardiovascular risk factors, may exacerbate plaque instability and precipitate acute coronary syndrome. This clinical scenario merits heightened clinical vigilance, particularly in patients with subclinical coronary atherosclerosis, yet relevant clinical case reports remain scarce.

## 2. Case Report

A 58-year-old female patient presented to the emergency department of our hospital with complaints of chest pain. The patient reported experiencing sudden, persistent, substernal squeezing pain without an identifiable trigger, accompanied by profuse sweating, persisting for over five hours without alleviation. An electrocardiogram (ECG) obtained at an external facility revealed ST-segment elevation in the inferior leads (III, aVF), and cardiac biomarker analysis demonstrated elevated troponin T levels (0.056 ng/mL). A diagnosis of acute ST-segment elevation myocardial infarction (STEMI) was established, and the patient received a loading dose of aspirin and a P2Y12 receptor antagonist prior to transfer to our hospital for further management.

**Figure 1:**
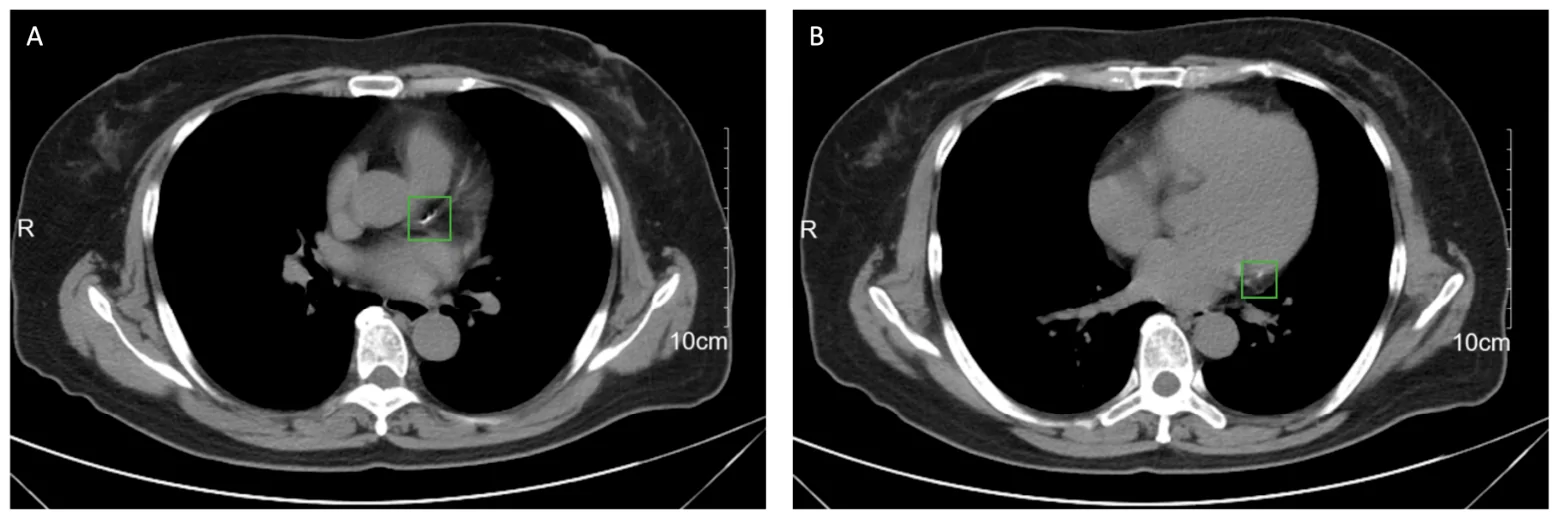
Preoperative chest-computed tomography. (**A**) Calcification in the left anterior descending artery (green box). (**B**) Calcification in the left circumflex artery (green box).

One year prior, the patient underwent left breast segmental resection and breast-conserving radical surgery at our institution. Postoperative pathological examination revealed invasive ductal carcinoma, with a pathological stage of pT1a(mi)N0M0. Immunohistochemical analysis indicated that the estrogen receptor and progesterone receptor were negative, HER2 was positive (3+), and Ki-67 was approximately 40%. The patient subsequently received four cycles of the THP regimen, consisting of docetaxel, pertuzumab, and trastuzumab, followed by eight cycles of dual HER2-targeted therapy with pertuzumab and trastuzumab, four cycles of trastuzumab monotherapy, and 29 fractions of adjuvant intensity-modulated radiotherapy to the left breast. At baseline, the patient was postmenopausal, with a BMI of 22.4 kg/m^2^, no history of hypertension, hyperlipidemia and diabetes, and no family history of cardiovascular disease. Hepatic and renal function, hemoglobin A1c level were normal. During anti-tumor therapies, mild hepatic insufficiency occurred, low-density lipoprotein cholesterol (LDL-C) level fluctuated from 3.03 mmol/L to a peak of 4.31 mmol/L, before decreasing to 2.89 mmol/L following ezetimibe therapy. Preoperative chest-computed tomography indicated coronary artery calcification (CAC) in the left anterior descending artery and the left circumflex artery, suggesting the presence of subclinical coronary atherosclerosis (Figure 1A,B). Acute STEMI manifested 17 days after the last maintenance anti-HER2 infusion, 223 days after radiotherapy completion, and 269 days after chemotherapy completion.

**Figure 2:**
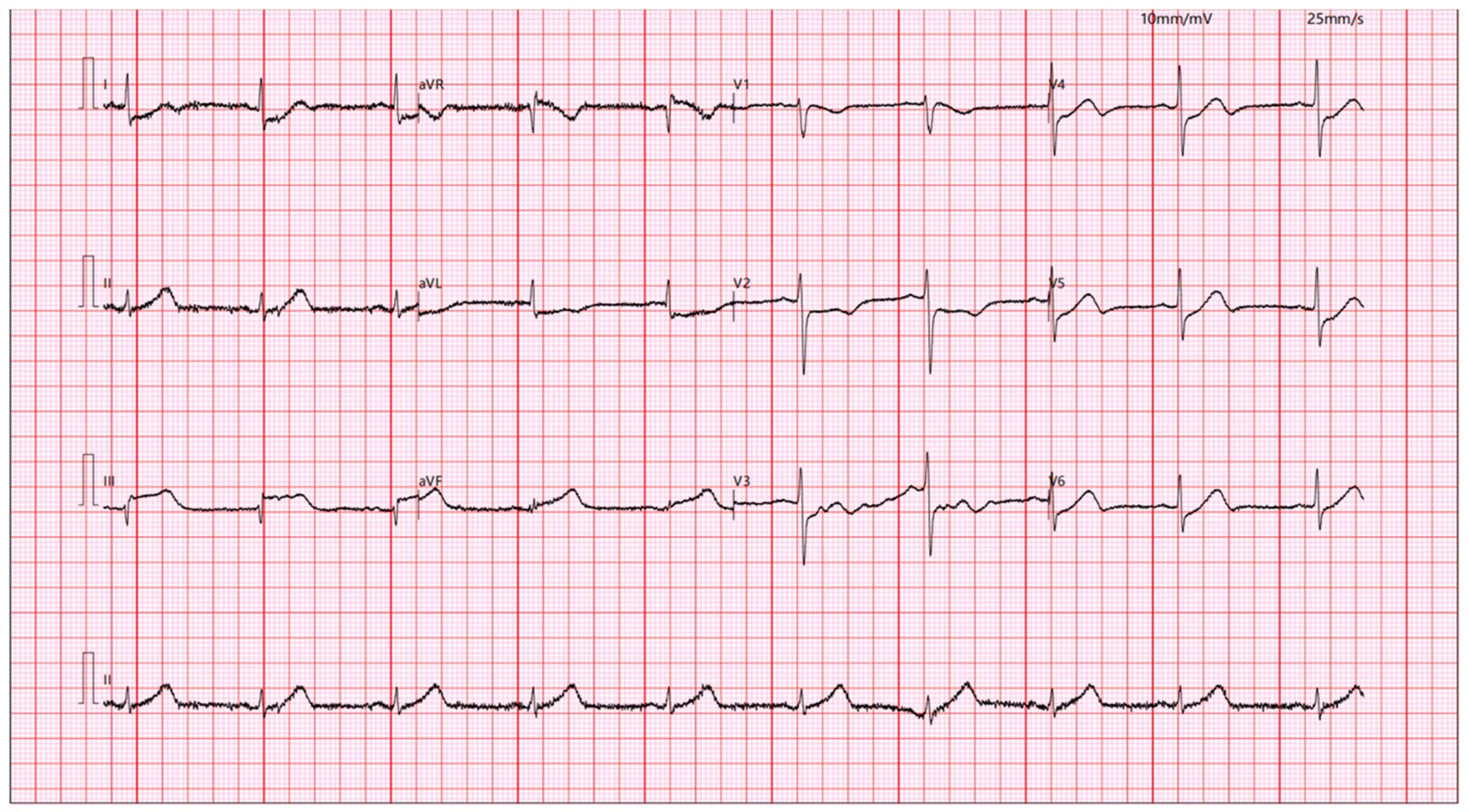
Electrocardiogram at admission showed sinus bradycardia, ST-segment elevation in the inferior leads, and ST-segment depression in V2–V6, I and aVL leads.

**Figure 3:**
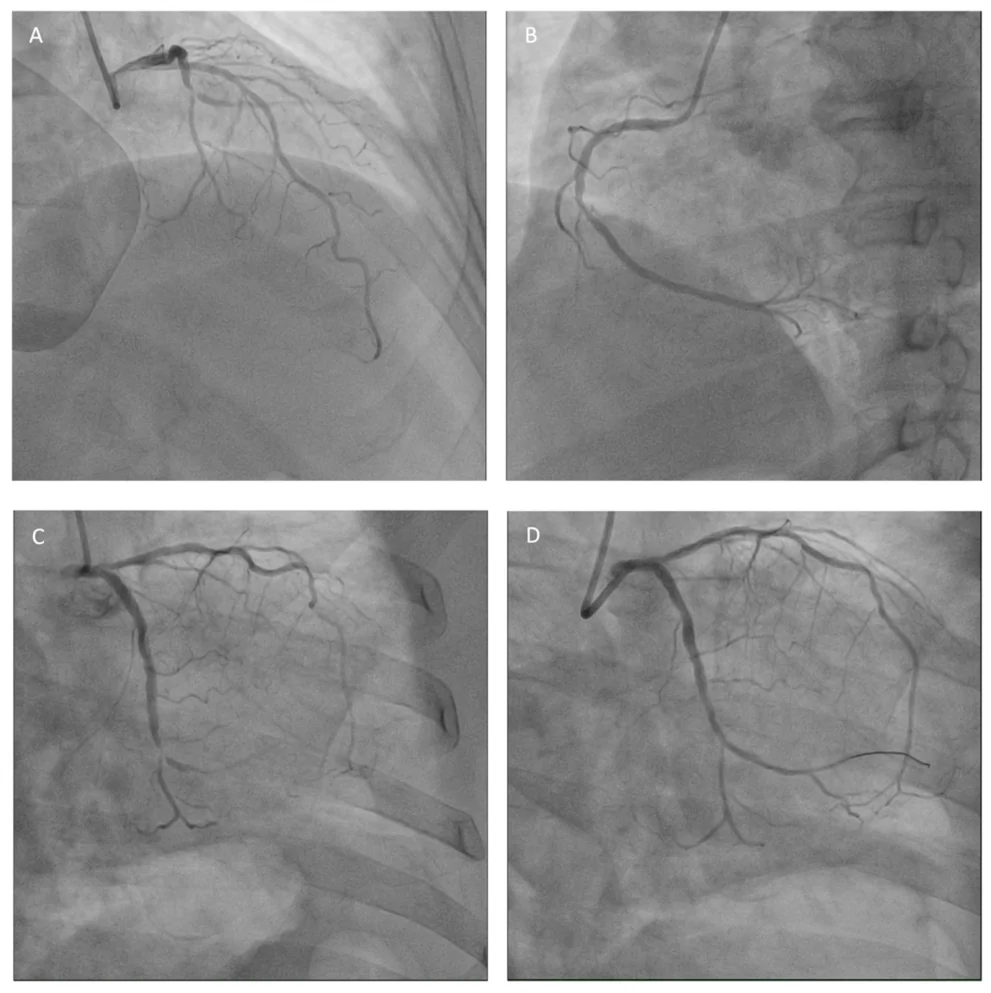
Coronary angiography (CAG). (**A**) CAG showed 50–60% stenosis in the proximal to middle segment of the left anterior descending artery. (**B**) CAG showed 50% stenosis in the middle segment of the right coronary artery. (**C**) Preoperative CAG showed subtotal occlusion in the middle segment of the left circumflex artery extending to the first obtuse marginal branch. (**D**) Post-procedural CAG showed that the coronary artery blood flow of the left circumflex-obtuse marginal lesion recovered well after drug-coated balloon angioplasty.

**Figure 4:**
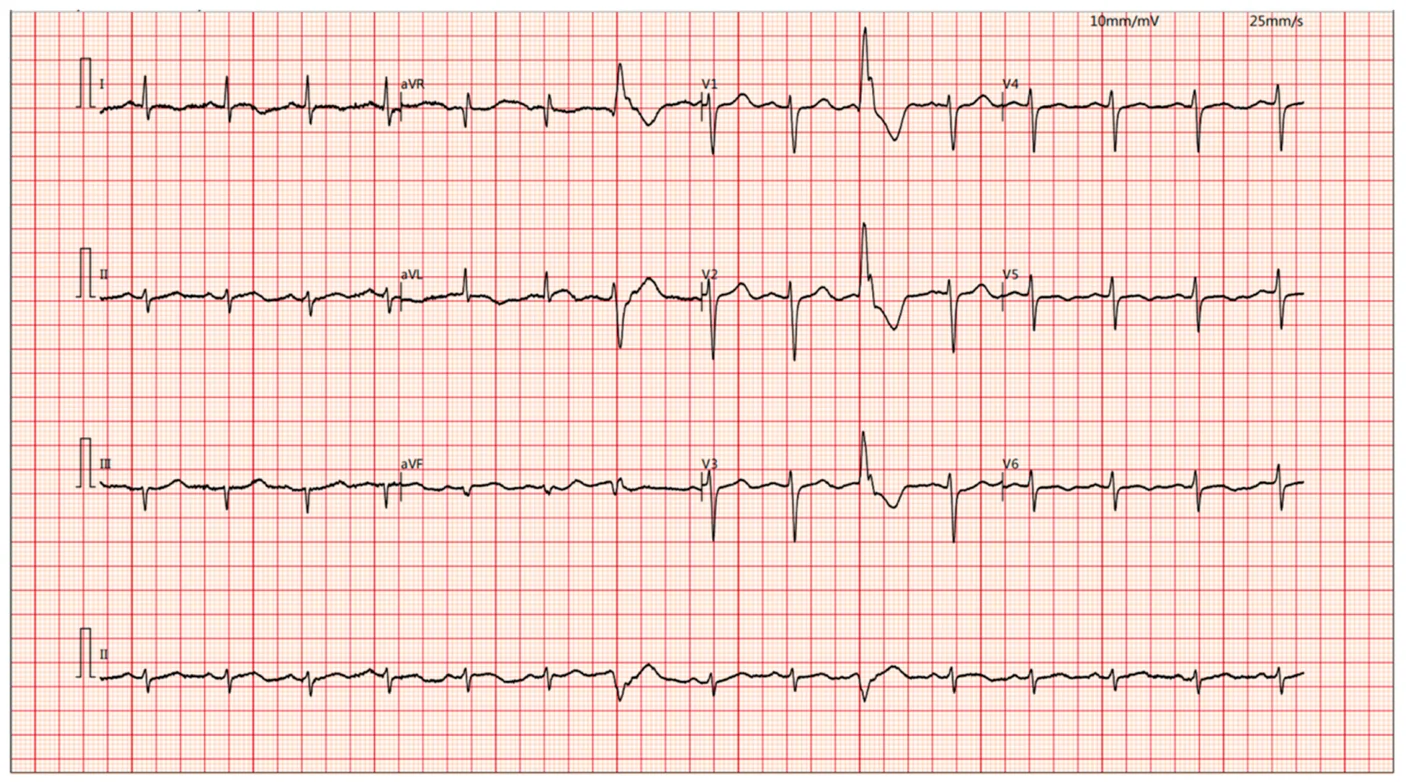
Post-procedural electrocardiogram showed sinus rhythm, premature ventricular contractions, and T-wave inversions in the I, aVL, V5, and V6 leads.

**Figure 5:**
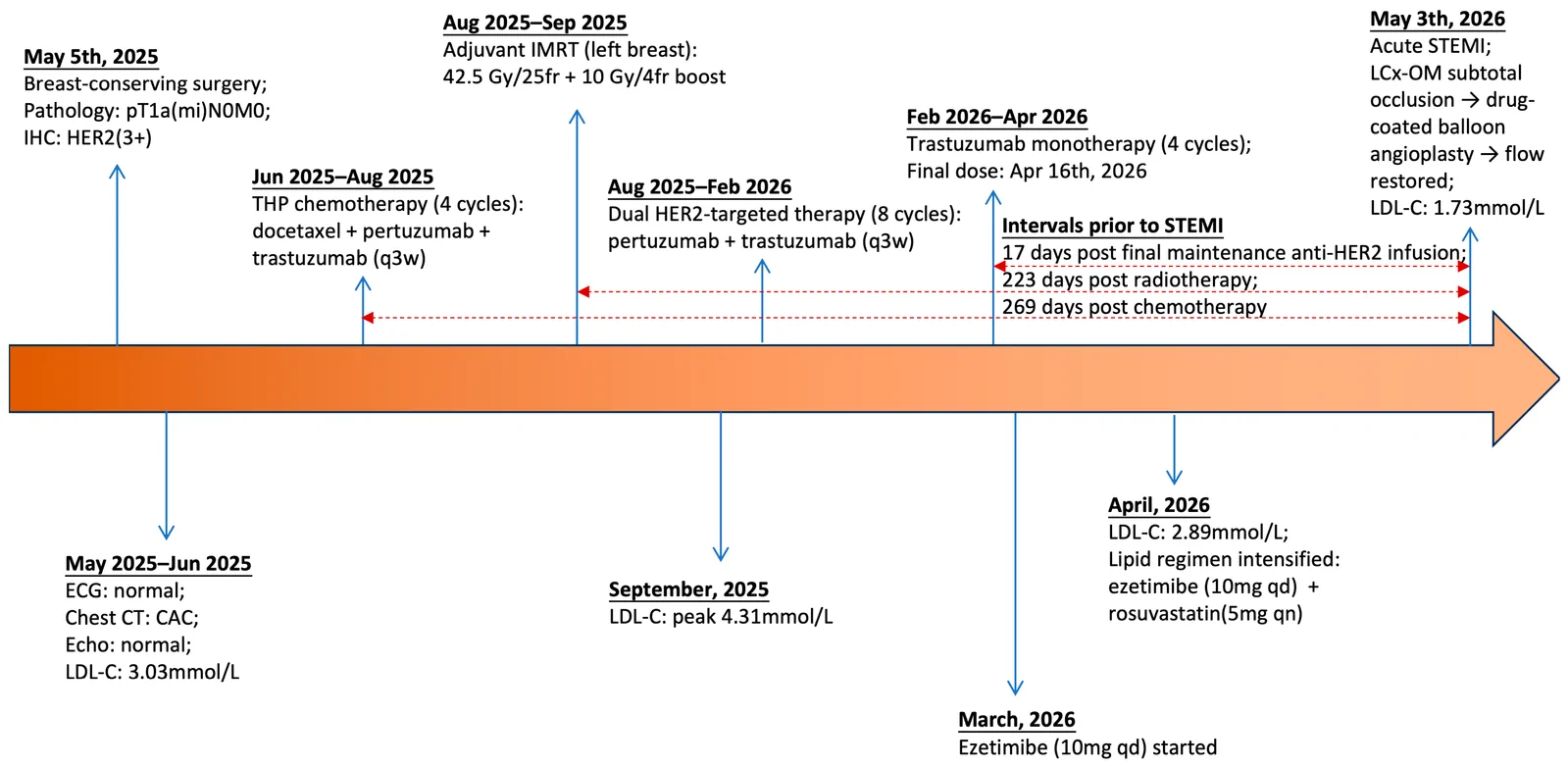
Timeline of anti-tumor therapies and follow-up. HER2: Human epidermal growth factor receptor 2; IHC: Immunohistochemistry; THP: Docetaxel, pertuzumab, and trastuzumab; IMRT: Intensity-Modulated Radiation Therapy; Gy: Gray; fr: Fraction; q3w: Every 3 weeks; ECG: Electrocardiogram; CT: Computed tomography; CAC: Coronary artery calcification; Echo: Echocardiography; LDL-C: Low-density lipoprotein cholesterol; STEMI: ST-segment elevation myocardial infarction; LCx: Left circumflex artery; OM: Obtuse marginal branch; Jun: June; Aug: August; Sep: September; Feb: February; Apr: April. qd: Every day; qn: Every night.

Her vital signs at admission were a temperature of 36.5 °C, blood pressure of 102/64 mmHg, a respiration rate of 15 breaths per minute, and a pulse rate of 96 beats per minute. Cardiopulmonary examination was unremarkable. Abdominal and neurological examinations revealed no abnormalities. Cardiac biomarkers showed elevated troponin-T (0.066 ng/mL) and troponin I (0.06 ng/mL), but normal creatine kinase-MB and B-type natriuretic peptide. ECG at admission indicated sinus bradycardia, ST-segment elevation in the inferior leads, and ST-segment depression in V2–V6, I and aVL leads (Figure 2). She was initially diagnosed with acute STEMI and then immediately sent for coronary angiography. The angiographic findings revealed 50–60% stenosis in the proximal to middle segment of the left anterior descending artery, 50% stenosis in the middle segment of the right coronary artery, and subtotal occlusion in the middle segment of the left circumflex artery extending to the first obtuse marginal branch, identified as the culprit lesion (Figure 3A–C). Consequently, drug-coated balloon angioplasty was promptly performed on the left circumflex-obtuse branch lesion, resulting in the satisfactory restoration of coronary artery blood flow post-procedure (Figure 3D). Due to the priority of emergent revascularization for acute STEMI, intravascular ultrasound and optical coherence tomography examinations were omitted. The post-procedural ECG indicated sinus rhythm, premature ventricular contractions, and T-wave inversions in the leads I, aVL, V5, and V6 (Figure 4). Echocardiography demonstrated regional wall motion abnormalities in the left ventricle, and normal global left ventricular systolic function. During hospitalization, the patient did not experience heart failure, malignant arrhythmias, or other significant complications. Following treatment with dual antiplatelet therapy, intensive lipid-lowering therapy, and additional symptomatic supportive measures, the patient exhibited a favorable recovery, with resolution of chest pain, and was discharged without incident. The patient continued to receive coordinated oncological and cardiovascular follow-up care (Figure 5).

## 3. Discussion

HER2-targeted therapies have significantly improved survival in patients with early-stage breast cancer, but cardiovascular toxicity remains a critical factor influencing prognosis [1]. Although cardio-oncology has driven improvements in risk assessment systems [7], current risk stratification still focuses primarily on cardiac dysfunction [8]. Conversely, the identification of risks associated with ischemic cardiovascular events, particularly in patients with subclinical atherosclerosis, remains comparatively inadequate. This case highlights the need to consider vascular toxicity related to anti-tumor therapies in clinical practice.

Accumulating evidence has confirmed that multimodal anti-tumor therapies induce broad vascular toxicity beyond myocardial injury. For trastuzumab and other HER2-targeted therapies, cardiotoxicity is more commonly characterized by myocardial dysfunction and reduced left ventricular ejection fraction. Nevertheless, Terwoord et al. have reported persistent microvascular endothelial dysfunction in patients treated with doxorubicin and/or trastuzumab, suggesting that vascular abnormalities may also accompany HER2-targeted therapies [4]. Mechanistic evidence for docetaxel-related vascular toxicity is also emerging. Docetaxel has been reported to impair endothelial function in human vessels [3,6]. These findings provide biological support for vascular effects of HER2-targeted therapies and taxanes. Chemotherapy and radiotherapy promote ischemic events via multiple mechanisms—endothelial dysfunction, inflammation, vasospasm, and thrombosis—that accelerate atherosclerosis and precipitate plaque rupture [5,9,10]. Collectively, these findings support a potential association between anti-tumor therapies and ischemic cardiovascular events, suggesting that anti-tumor therapy may act as an additional vascular stressor in patients with pre-existing coronary atherosclerosis.

Importantly, the 2022 European Society of Cardiology Guidelines on cardio-oncology advocates cardiovascular risk assessment before anti-tumor therapy and risk factor management during treatment [11]. Detection of CAC on baseline pre-antineoplastic chest-computed tomography should prompt clinicians to initiate early cardiovascular risk stratification and primary prevention [10,11,12]. Whether earlier intensive lipid-lowering after CAC detection in this case would have altered outcomes is uncertain. Notably, the patient’s LDL-C progressively climbed from 3.03 mmol/L to a peak of 4.31 mmol/L during multimodal anti-tumor care. Even though short-term lipid-lowering intervention lowered LDL-C to 1.73 mmol/L, acute STEMI still developed. Coronary angiography revealed multivessel atherosclerotic lesions, indicating that brief lipid-lowering treatment fails to rapidly stabilize or reverse longstanding atherosclerotic plaque burden.

In this case, multimodal anti-tumor therapies, including docetaxel-based chemotherapy, left-sided breast radiotherapy, and HER2-targeted therapies, may serve as a vascular toxic “second-hit”. These interventions accelerate the progression or destabilization of pre-existing coronary lesions, instead of triggering de novo coronary artery disease in isolation. Meanwhile, this case highlights that cardiovascular surveillance during HER2-targeted therapies should extend beyond left ventricular function to include vascular risk assessment and preventive management, particularly when CAC is identified at baseline.

## 4. Conclusions

This case underscores the importance for clinicians to recognize that patients presenting with CAC or other indicators of subclinical atherosclerosis may be at an elevated risk for ischemic cardiovascular events when undergoing multimodal anti-tumor therapies. Consequently, it is imperative to conduct more proactive risk assessments for ischemic events, implement comprehensive risk factor management, and ensure sustained cardiovascular monitoring throughout the course of anti-tumor treatment and subsequent survivorship care. Early detection of CAC, coupled with comprehensive cardio-oncology management, has the potential to enhance long-term cardiovascular outcomes in high-risk patient populations.

## Data Availability

The data presented in this study are available on request from the corresponding author due to reasons of sensitivity.

## Abbreviations

The following abbreviations are used in this manuscript:

HER2	Human epidermal growth factor receptor 2
ECG	Electrocardiogram
STEMI	ST-segment elevation myocardial infarction
CAC	Coronary artery calcification
LDL-C	Low-density lipoprotein cholesterol
